# The Stability of Intercalated Sericite by Cetyl Trimethylammonium Ion under Different Conditions and the Preparation of Sericite/Polymer Nanocomposites

**DOI:** 10.3390/polym11050900

**Published:** 2019-05-17

**Authors:** Yu Liang, Dexin Yang, Tao Yang, Ning Liang, Hao Ding

**Affiliations:** 1 School of Materials Science and Technology, Shenyang University of Chemical Technology, Shenyang 110142, China; liangyuaadd@126.com; 2 Beijing Key Laboratory of Materials Utilization of Nonmetallic Minerals and Solid Wastes, National Laboratory of Mineral Materials, School of Materials Science and Technology, China University of Geosciences (Beijing), Beijing 100083, China; liangning628@126.com; 3 College of Materials and Environmental Engineering, Hangzhou Dianzi University, Hangzhou 310018, China; dy263@hdu.edu.cn (D.Y.); yangtao@hdu.edu.cn (T.Y.)

**Keywords:** layered silicate, sericite, CTAB, intercalation stability, nanocomposites

## Abstract

Layered silicates are suitable for use as fillers in nanocomposites based on their particular features, such as large aspect ratio, easy availability, and chemical resistance. Among them, sericite is distinguished for its higher aspect ratio, higher resilience, and ultraviolet shielding and absorption. Previously, sericite was structure-modified and intercalated by CTAB to expand its interlayer space. The intercalated sericite seems promising for use in the fabrication of polymer/sericite composites or pillared sericite. However, special attention should be paid to the stability of the intercalated sericite because the CTAB inside the layer may be de-intercalated, which would affect the interlayer spacing and its surface properties. In this article, the stability of the sericite intercalated by CTAB was tested by changing different variables, such as different washing solvents, different temperatures, ultrasonic cleaning, and different solution conditions. Finally, sericite/polymer nanocomposites were produced with the stable intercalated sericite, and showed excellent properties compared with pure epoxy resin.

## 1. Introduction

Recently, nanocomposites have attracted great attention due to their extraordinary properties. A wide variety of fillers, either natural ones or synthetic ones, can be used to intercalate into the polymer [1,2]. The conventional fillers for polymers are inorganic materials of mainly three categories: particles (e.g., calcium carbonate particles), fibers (e.g., glass fibers and carbon fibers), and plate-shaped particles (e.g., montmorillonite and vermiculite) [3,4,5,6,7,8]. Among them, layered silicates attract the most attention because of their easy availability, large aspect ratios, and chemical resistance [9,10,11,12]. However, in most cases, these layered silicates, which are hydrophilic by nature, need to be organically modified to produce polymer-compatible clay, to increase the compatibility between the layered silicate and the polymer matrix, and to lower the surface energy of the layered silicate. Until now, many methods have been developed to modify layered silicate. For example, the replacement of the inorganic exchange cations with alkylammonium surfactants can make clay compatible to the polymer matrix [13,14,15].

Many layered silicates can be organically modified for the fabrication of polymer/layered silicate nanocomposites. Some commonly used substrates for intercalation are swelling clays, such as kaolinite [16,17,18], vermiculite [19,20,21,22], and montmorillonite [23,24,25]. A few articles use sericite as a raw material for the fabrication of polymer/layered silicate nanocomposites. Sericite is a kind of 2:1 phyllosilicate similar to muscovite with a series of unique properties. It has a higher aspect ratio (more than 1000) [26,27,28], and higher resilience than other fillers, and can shield and absorb ultraviolet radiation [29]. Unlike other 2:1 phyllosilicates such as montmorillonite and vermiculite, sericite cannot swell in water, and is non-expandable due to the substitution of Al^3+^ for Si^4+^ in tetrahedral sheets, which results in a net negative surface charge balanced by K^+^, filling in the interlayer tightly. Therefore, sericite needs to be modified first in order to allow water and other molecules to enter the interlayer, and for cation exchanges. Previously, our team has successfully modified sericite by thermal modification, acid activation, and sodium modification [30]. The cation exchange capacity of the modified sericite reached 56.37 mmol/100 g, making it a suitable material for organic modification. The modified sericite was then intercalated by CTAB to expand its interlayer space from 1.00 nm to 5.07 nm. Therefore, sericite seems promising for use in the next step—for the fabrication of polymer/sericite composites or pillared sericite (e.g., Al-pillared sericite) in order to obtain better performances.

As for the intercalation of layered silicate, a lot of attention is given to methods that can modify layered silicate from hydrophilic to hydrophobic, but not much attention has been given to the stability of the modified layered silicate (or intercalated layered silicate). However, it should be noted that the stability of the intercalated layered silicate is very important, since the intercalated silicate needs to be used in the next step—for the fabrication of clay–polymer nanocomposites, or for the fabrication of pillared silicates (such as TiO_2_ pillared montmorillonite). If the intercalated layered silicate is not very stable, the interlayer spacing of the layered silicate will be changed, and its surface properties will be affected. Gu et al. [31] found that part of the intercalated CTAB in montmorillonite was de-intercalated when the colloidal particles produced by the hydrolysis of tetrabutyl titanate were in the suspension. The content of CTA^+^ in the interlayer decreased, and the distribution and inclination angle of CTA^+^ in the interlayer changed accordingly. Therefore, there is a great necessity to study the stability of intercalated layered silicate.

In this article, the stability of intercalated sericite was researched. Raw sericite (S0) was first modified to gain cation exchange capacity by thermal modification, acid activation, and sodium modification. Then, the modified sericite was intercalated by CTAB to increase d-space and change its surface wettability. Different conditions (e.g., washing solvents with different temperatures) were used to test the stability of the intercalated sericite. Finally, the stable intercalated sericite was added into epoxy resin to produce sericite/polymer nanocomposites, and these showed excellent properties. This article aims to provide some guidance to those who use intercalated layered silicate for the fabrication of polymer/layered silicate nanocomposites or pillared silicate.

## 2. Materials and Methods

### 2.1. Materials

**Figure 1:**
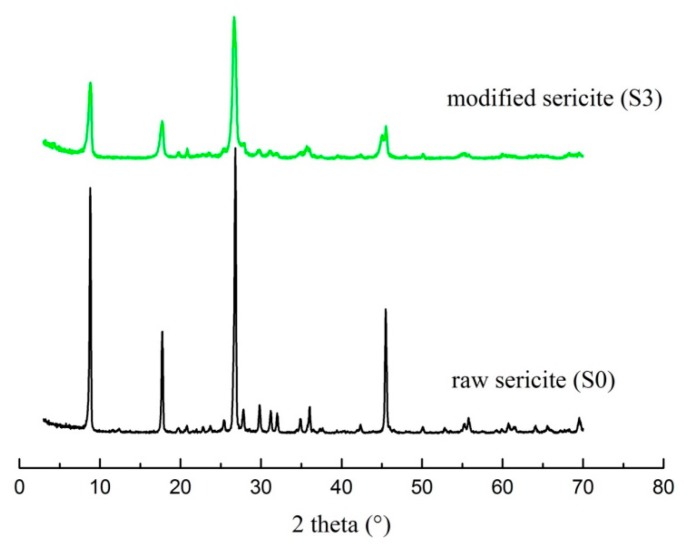
XRD patterns of S0 and S3.

**Figure 2:**
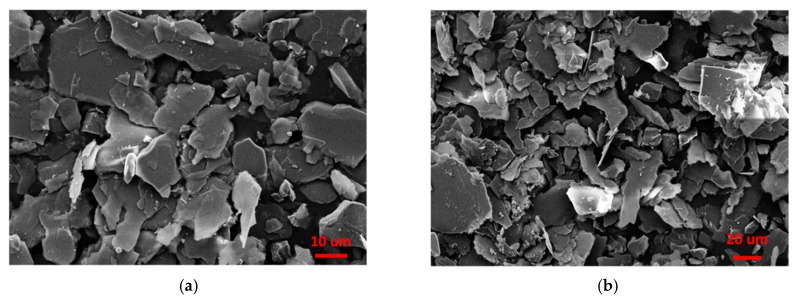
SEM images of (**a**) S0 and (**b**) S3.

Following gravity purification, drying, and grinding, raw sericite materials (S0) were obtained from the natural sericite produced in Anhui, China. S1 was produced by heating raw sericite materials at 800 °C for 1 h, and cooling them down to room temperature. S2 was produced by mixing 6 g of the thermally treated sericite with 200 g 5 M nitric acid at 95 °C for 4 h in a water bath. Stirring at a speed of 150 rpm and drying at ambient temperature was required for the next modification step. S3 was obtained by mixing 6 g of S2 with 200 g of saturated NaCl solution, and by stirring at the same rotational speed as before at 95 °C for 3 h. Then, S3 was washed with DI H_2_O and dried at room temperature. The cation exchange capacity (CEC) value of S3 reached 56 mmol/100 g, which is a great improvement compared to raw sericite (7.5 mmol/100 g). The XRD patterns and SEM images of S0 and S3 are shown in Figure 1 and Figure 2, respectively. All chemicals were of analytical grade, and were used without further purification.

### 2.2. The Intercalation Process and the Stability Tests

In order to test the stability of the intercalated product under different washing conditions (different solvents and temperatures), S3 was intercalated with CTAB (the amount of CTAB was 15 times the CEC of S3) at 80 °C for 24 h in a water bath. Then the product was washed with hot DI H_2_O (50 °C), cold DI H_2_O (25 °C), and ethanol (25 °C) separately, then centrifuged and dried. Ultrasonic cleaning was used in the procedure of washing to see if it had a negative effect on the stability of the intercalated product. The stability of intercalated product in the solution was also tested by putting the intercalated product in DMSO, and in a solution of CTAB (DMSO was used as solvent, and the amount of CTAB was 15 times theCEC of S3) at 80 °C for 24 h in a water bath, separately. Finally, different substrates were used in the intercalation process to see which one was better for future use.

### 2.3. The Preparation Process of Sericite/Polymer Nanocomposites

**Figure 3:**
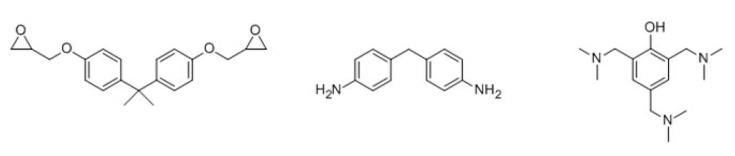
The chemical structure of diglycidylether of bisphenol A (DGEBA) resin (**left**), 4,4-diaminodiphenyl methane (DDM) hardener (**middle**), and tris(dimethylaminomethyl)phenol (DMP-30) accelerator (**right**).

The epoxy-based matrix consists of a two functional diglycidylether of bisphenol A (DGEBA, Hubei Jusheng Technology, Wuhan, China) which is cured stoichiometrically with 4,4-diaminodiphenyl methane (DDM, Changsha Jiazhen biological Company, Changsha, China). As an accelerator, 3 wt.% tris(dimethylaminomethyl)phenol (DMP-30, Changzhou Shanfeng Chemical, Changzhou, China) was added to the reactive mixture. All nanocomposites were then cured at 80 °C for 2 h, and then at 150 °C for 2 h to achieve a fully cured polymer matrix. The chemical structures of the reactive components are presented in Figure 3.

### 2.4. Characterization

The changes in the d_001_ value of the sericite were determined by X-ray diffraction (XRD) analyses. The percentage of the intercalated layers to the total layers, defined as the intercalation rate, was used to judge the degree of intercalation. The XRD analyses were conducted with a Rigaku D/max-rA (12 kW) X-ray powder diffractometer (Rigaku, Tokyo, Japan), operated with Cu Kα radiation at 40 kV and 100 mA, and with a scanning speed of 0.5° (2θ)/min. The microstructure and morphologies were investigated by a Hitachi S-3500 SEM (Hitachi, Tokyo, Japan). The morphologies of the sericite/epoxy nanocomposites were characterized using a Tecnai F-20 transmission electron microscope (FEI, Hillsboro, OR, USA), applying a 200 kV accelerating voltage. The mechanical properties of the sericite/epoxy nanocomposites were tested by an electronic universal testing machine (WDW-300, Shimadzu, Kyoto, Japan).

## 3. Results and Discussion

### 3.1. The Effect of Different Washing Solvents and Temperatures on the Stability of the Intercalated Product

**Figure 4:**
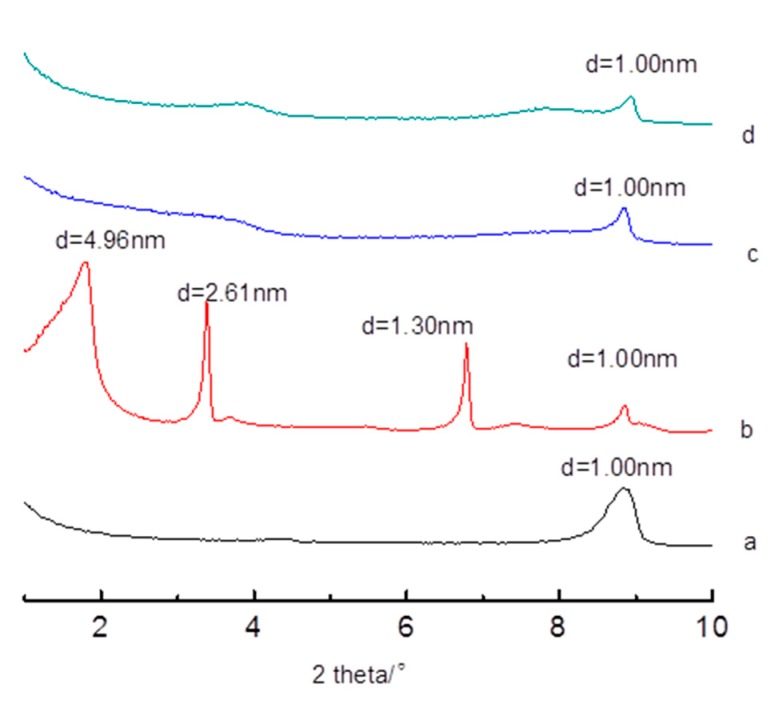
XRD patterns of the intercalated products washed with different solvents at different temperatures. **a**. S3; **b**. the intercalated product washed with cold DI H_2_O (25 °C); **c**. the intercalated product washed with hot DI H_2_O (50 °C); **d**. the intercalated product washed with cold ethanol (25 °C).

Figure 4 shows the XRD patterns of the intercalated products washed with different solvents at different temperatures. Obviously, cold DI H_2_O had the best effect of keeping the intercalation result. After being washed with cold DI H_2_O, the d-value of the newly emerged diffraction peak was 4.96 nm, which was more than five times the d-value of the modified sericite (S3). The peak pattern was sharp as well as symmetric, which means CTAB had ordered arrays in the interlayer of sericite. While the effects of hot DI H_2_O and cold ethanol were much worse than cold DI–H_2_O, no obvious diffraction peaks could be seen in the two XRD patterns. This means that washing with hot DI H_2_O and ethanol can make the CTAB in the interlayer de-intercalate and arrange in a disorderly manner. In spite of the de-intercalated effect, the final product still had some intercalated effect compared to S3.

CTAB has a much higher solubility in hot water and in ethanol than in cold water, which caused the de-intercalation of CTAB in the interlayer of sericite. Therefore, cold DI H_2_O should be used during the washing procedure in order to guarantee the modification effect.

### 3.2. The Effect of Ultrasonic Cleaning on the Stability of the Intercalated Product

**Figure 5:**
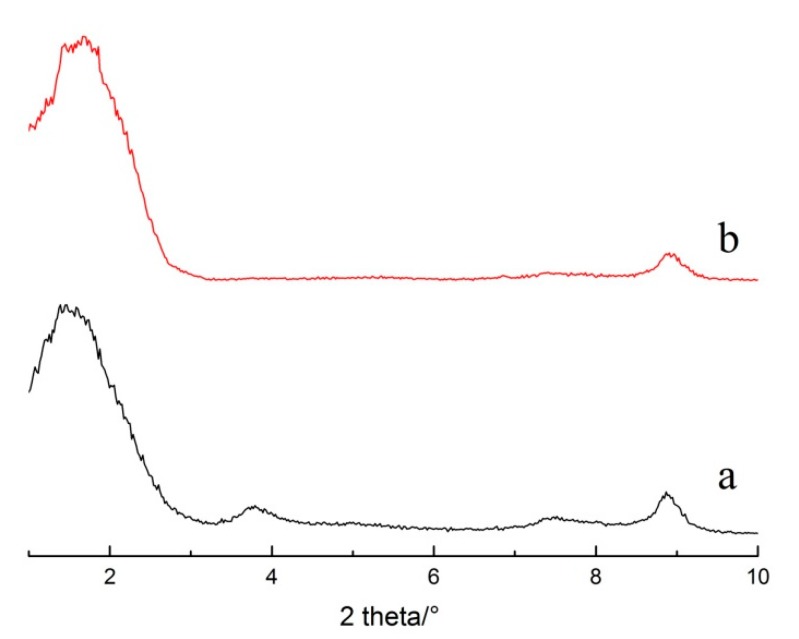
XRD patterns of the intercalated products: **a**. washed with ultrasonic cleaning; **b**. washed without ultrasonic cleaning.

Figure 5 shows the XRD patterns of the intercalated products after being treated with and without ultrasonic waves during the washing procedure. The solvent used in the washing procedure was cold DI H_2_O. It can be seen that ultrasonic cleaning had no obvious effect on the intercalation result.

### 3.3. The Stability of the Intercalated Product in the Solution

**Figure 6:**
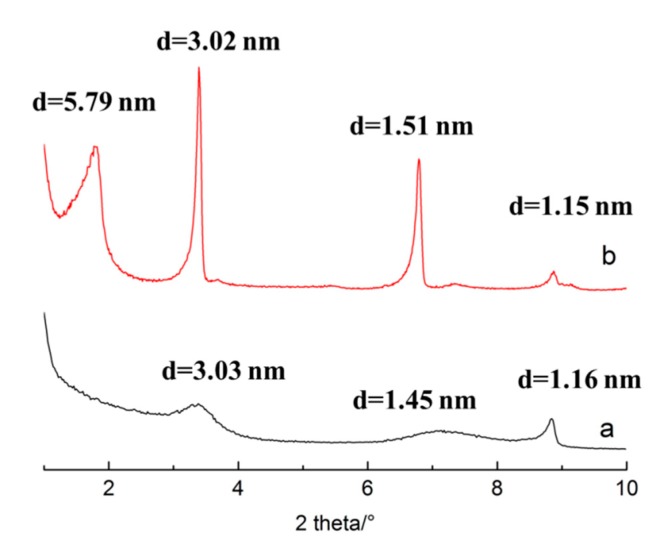
Stability test of the intercalated products in the solution: **a**. the intercalated sericite was used as raw material and DMSO was used as a solvent, at 80 °C for 24 h; **b**. the intercalated sericite was used as raw material, CTAB (the amount of CTAB was 15 times the cation exchange capacity (CEC) of S3) and DMSO were used in the solution at 80 °C for 24 h.

Intercalation is not the final procedure in the fabrication of polymer/clay nanocomposites or pillared composites. The intercalated products need to be used as raw material for the next step. Therefore, it is necessary to keep the intercalated product stable in the solution. In this part, the stability of intercalated product in the solution was tested by putting the intercalated product in DMSO solvent, and in a solution of CTAB (DMSO was used as the solvent and the amount of CTAB was 15 times the CEC of S3) at 80 °C for 24 h in a water bath, separately. Figure 6 shows the XRD patterns of the final products. The d-space of the intercalated sericite in the mixed solution of CTAB and DMSO was 5.79 nm, and the peak patterns were sharp, which means good crystallinity of the final product. The d-space of the intercalated sericite in the DMSO only reached 3.03 nm, and the diffraction peaks were much flatter. Therefore, it is clear that the stability of the intercalated product in the mixed solution of CTAB and DMSO was much better than in DMSO.

A hot solvent such as DMSO can dissolve CTAB and move CTAB out from the interlayer, causing the disorder of CTAB in the interlayer. However, the mixed solution of CTAB and DMSO can ensure a saturated solution of CTAB (the amount of CTAB was 15 times the CEC of S3, much higher than the solubility of CTAB in this temperature), and maintain the stability of CTAB in the interlayer with the help of osmotic pressure. Therefore, if the intercalated sericite needs to be used in a subsequent step, sufficient CTAB should be put into the solution in order to keep the intercalated CTAB stable inside the interlayer.

### 3.4. The Choice of Raw Material for the Intercalation Based on Stability, Intercalation Rate, and Other Experimental Conditions

**Figure 7:**
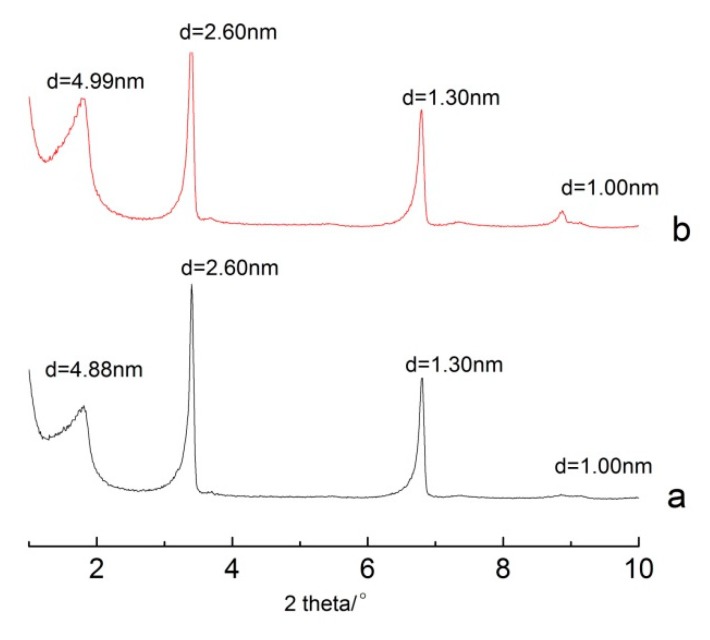
XRD patterns of different raw materials used for the intercalation: **a**. raw material was S3, CTAB was used as intercalation agent in DMSO solvent; **b**. raw material was intercalated S3, CTAB was used as intercalation agent in DMSO solvent.

Figure 7 shows the XRD patterns of different raw materials used in the intercalation. When S3 was used as a raw material, CTAB was used as an intercalation agent in DMSO solvent at 80 °C for 24 h. When the intercalated sericite was used as a raw material, CTAB was used as an intercalation agent in the DMSO at 80 °C for 3 h. It can be seen that it did not matter what the raw material was, the intercalation result was almost the same. The d-space was maintained between 4.88 and 4.99 nm, and both peak patterns were sharp and symmetrical.

The intercalation rates were calculated, and are shown in Table 1. From Table 1, it can be seen that both the intercalation rates were relatively high, ranging from 94.83% to 98.20%.

The usage of reagents and the experiment time are listed in Table 2. Obviously, the intercalated S3 consumed more reagent and had a longer experiment time. Based on these factors, S3 should be chosen for the intercalation. The intercalation did not improve by repeating it. The intercalated product made from S3 was stable, consumed less reagent, took less time, and had a higher intercalation rate.

### 3.5. Properties of the Sericite/Polymer Nanocomposites

**Figure 8:**
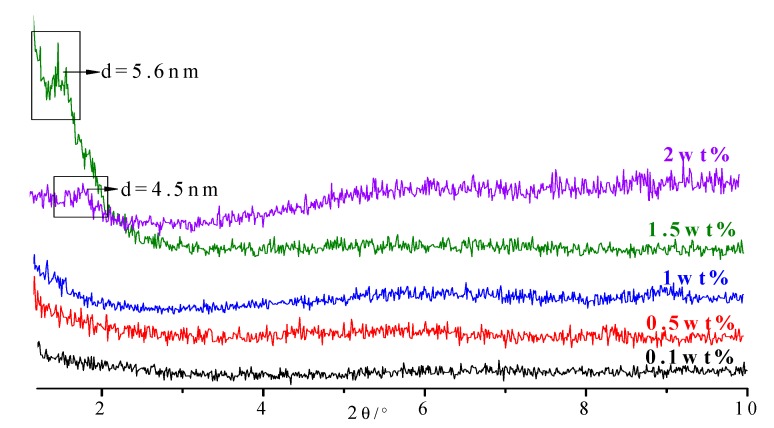
XRD patterns of the sericite/epoxy resin with different amounts of intercalated sericite added.

Different amounts of stable intercalated sericite, using the optical conditions mentioned above (0.1 wt%, 0.5 wt%, 1 wt%, 1.5 wt%, and 2 wt%), were added into epoxy resin to produce sericite/epoxy nanocomposites. In Figure 8, it can be seen that when the amount of sericite was less than 1 wt%, mainly exfoliated nanocomposites were formed. The interlayer spacing could be expanded to more than 8.8 nm, with the sheets of sericite distributed uniformly in the matrix. As the amount of sericite increased to 1.5 wt%, diffraction peaks emerged at 2θ < 2°, which means that part of the sericite was not exfoliated in the epoxy resin matrix, while the interlayer spacing still increased substantially. The increased amount of sericite increased the background of the previous d002 value of sericite, which means the exfoliation is not that completed, with part of sericite lamellar still in order degree.

**Figure 9:**
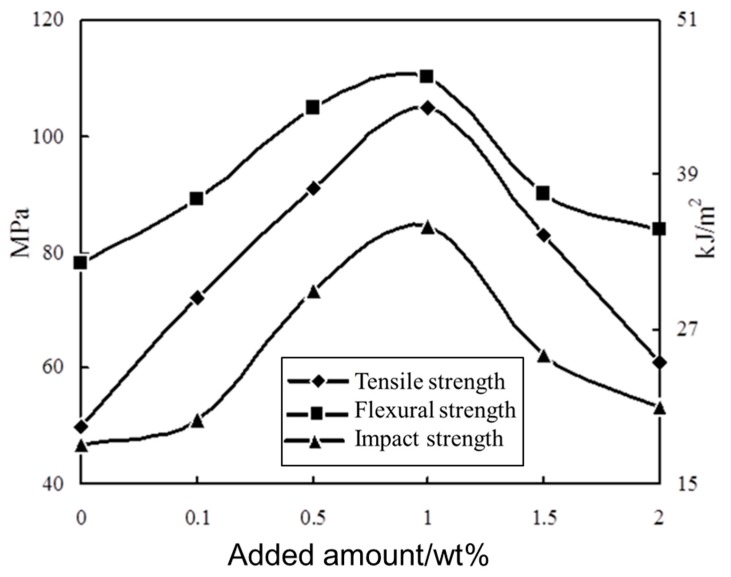
The effect of the added amount of intercalated sericite on the mechanical properties of the composites.

The tensile strength, flexural strength, and impact strength of the sericite/epoxy nanocomposites with different amounts of sericite added were also tested and compared with pure epoxy resin with no sericite added. In Figure 9, it can be seen that the mechanical strength of the nanocomposites increased substantially compared with pure epoxy resin. The mechanical strength was best when the amount of sericite was 1%. The tensile strength (105 MPa) increased by 110% compared with pure epoxy resin (50 MPa), the flexural strength (110 MPa) increased by 41% compared with pure epoxy resin (78 MPa), and the impact strength (35 kJ/m^2^) increased by 94% compared with pure epoxy resin (18 kJ/m^2^).

**Figure 10:**
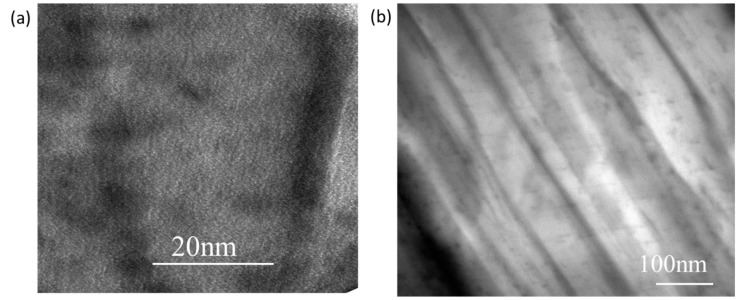
TEM images of the sericite/epoxy nanocomposites (the amount of sericite was 1 wt %). (**a**) scale bar 20 nm; (**b**) scale bar 100 nm.

Figure 10 shows TEM images of the sericite/epoxy nanocomposites (the amount of sericite was 1 wt%). The dark textures are sericite layers, while the light parts are epoxy resin. The sericite interlayers were still parallel while most of them were exfoliated to a relatively large extent with the distance at 20 nm or more. Together with the XRD patterns, this result proves the formation of exfoliated sericite/epoxy nanocomposites.

When compared with virgin epoxy resin, the sericite/epoxy resin nanocomposites had better mechanical properties. The results above also suggest that there is an optimum clay concentration for the nanocomposite, which was 1% in our case. Compared with epoxy resin, sericite is more naturally resistant to straining due to its high modulus. Moreover, when the added amount of sericite was less than (or equal to) 1%, mainly exfoliated sericite/epoxy nanocomposites could be formed, with most of the sericite lamella distributed uniformly in the epoxy resin. Therefore, the mechanical properties of sericite/epoxy nanocomposites improved dramatically when compared with pure epoxy resin. As the amount of sericite increased, the relative amount of intercalation/exfoliation of the sericite gradually increased with an increase in the sericite content, which means that the dispersion of sericite in epoxy resin was not as good as before, leading to decreased mechanical properties. The modifier of sericite—CTAB in our case—helped change the surfaces of sericite from hydrophilic to hydrophobic, leading to good adhesion between sericite and epoxy resin, which was the precondition to the improvement of the nanocomposite mechanical properties.

## 4. Conclusions

In summary, the intercalated sericite by cetyl trimethylammonium ion was stable during the washing procedure with cold (room-temperature) DI H_2_O. Hot DI H_2_O and ethanol could de-intercalate CTAB in the interlayer. Ultrasonic cleaning had no effect on the stability of the intercalated product. If the intercalated product needs to be used in a subsequent reaction step, a sufficient amount (larger than the solubility of CTAB) of CTAB should be put into the solution in order to prevent the de-intercalation of CTAB from the interlayer. The intercalated product made from S3 was stable, consumed less reagents, and had a higher intercalation rate compared with twice-intercalated products. The sericite/epoxy nanocomposites produced with stable intercalated sericite had better mechanical properties than pure epoxy resin. Mainly exfoliated sericite/epoxy nanocomposites with the layers of sericite dispersed very well in the epoxy resin were formed when the amount of sericite was 1 wt% or less based on the XRD and TEM results. Stable intercalated sericite is the precondition of a good adhesion between sericite and epoxy resin, which leads to good nanocomposite mechanical properties.

## Figures and Tables

**Table 1:** The intercalation rate using different raw materials.

	S3	Intercalated S3
**Intercalation rate (%)**	98.20	94.83

**Table 2:** The usage of reagents and experiment time with different raw materials for the intercalation.

	S3	Intercalated S3
**The amount of CTAB**	15 times the CEC of S3	30 times the CEC of S3
**Experiment time**	24 h	24 h + drying time + 3 h

## References

[1] Ha C.S. (2017). Polymer Based Hybrid Nanocomposites; A Progress Toward Enhancing Interfacial Interaction and Tailoring Advanced Applications. Chem. Rec..

[2] Alexandre M., Dubois P. (2000). Polymer-layered silicate nanocomposites: Preparation, properties and uses of a new class of materials. Mater. Sci. Eng. R Rep..

[3] Chen C., Xi J., Han Y., Peng L., Gao W., Xu Z., Gao C. (2018). Ultralight graphene micro-popcorns for multifunctional composite applications. Carbon.

[4] Ahn C., Kin S.-M., Jung J.-W., Park J., Kim T., Lee S., Jang D., Hong J.-W., Han S.M., Jeon S. (2018). Multifunctional polymer nanocomposites reinforced by 3D continuous ceramic nanofillers. ACS Nano.

[5] Hrachova J., Komadel P., Chodak I. (2009). Natural rubber nanocomposites with organo-modified bentonite. Clays Clay Miner..

[6] Tan X., Xu Y., Cai N., Jia G. (2009). Polypropylene/silica nanocomposites prepared by in-situ melt ultrasonication. Polym. Compos..

[7] Carvalho D., Carvalho J., Oliveira S., Rosa D. (2018). A new approach for flexible PBAT/PLA/CaCO3 film into agriculture. J. Appl. Polym. Sci..

[8] Mao Y., Shao C., Shang P., Li Q., He X., Wu C. (2019). Preparation of high strength PET/PE composites reinforced with continued long glass fibers. Mater. Res. Express.

[9] Chiu C.-W., Huang T.-K., Wang Y.-C., Alamani B.G., Lin J.-J. (2014). Intercalation strategies in clay/polymer hybrids. Prog. Polym. Sci..

[10] Ogawa M., Kuroda K. (1997). Preparation of Inorganic–Organic Nanocomposites through Intercalation of Organoammonium Ions into Layered Silicates. Bull. Chem. Soc. Jpn..

[11] Pavlidou S., Papaspyrides C.D. (2008). A review on polymer–layered silicate nanocomposites. Prog. Polym. Sci..

[12] Alateyah A.I., Dhakal H.N., Zhang Z.Y. (2013). Processing, Properties, and Applications of Polymer Nanocomposites Based on Layer Silicates: A Review. Adv. Polym. Technol..

[13] Jian X., Xuebing W., Bingyao D., Qingsheng L. (2016). Modification of montmorillonite by different surfactants and its use for the preparation of polyphenylene sulfide nanocomposites. High Perform. Polym..

[14] Xiong J., Liu Y., Yang X., Wang X. (2004). Thermal and mechanical properties of polyurethane/montmorillonite nanocomposites based on a novel reactive modifier. Polym. Degrad. Stab..

[15] Zhang H., Jia X., Yu J., Xue L. (2013). Effect of expanded vermiculite on microstructures and aging properties of styrene–butadiene–styrene copolymer modified bitumen. Constr. Build. Mater..

[16] Liang S., Li C., Dai L., Tang Q., Cai X., Zhen B., Xie X., Wang L. (2018). Selective modification of kaolinite with vinyltrimethoxysilane for stabilization of Pickering emulsions. Appl. Clay Sci..

[17] Frost R.L., Kristof J., Horvath E., Kloprogge J.T. (1999). Deintercalation of dimethylsulphoxide intercalated kaolinites: A DTA/TGA and Raman spectroscopic study. Thermochim. Acta.

[18] Tchoumene R., Dedzo G.K., Ngameni E. (2018). Preparation of Methyl Viologen-Kaolinite Intercalation Compound: Controlled Release and Electrochemical Applications. ACS Appl. Mater. Interfaces.

[19] Isci S., Isci Y. (2018). Characterization and comparison of thermal & mechanical properties of vermiculite polyvinylbutyral nanocomposites synthesized by solution casting method. Appl. Clay Sci..

[20] Isci S. (2017). Intercalation of vermiculite in presence of surfactants. Appl. Clay Sci..

[21] Su X., Ma L., Wei J., Zhu R. (2016). Structure and thermal stability of organo-vermiculite. Appl. Clay Sci..

[22] Wu N., Wu L., Liao L., Lv G. (2015). Organic intercalation of structure modified vermiculite. J. Colloid Interface Sci..

[23] Hattab Y., Benharrats N. (2015). Thermal stability and structural characteristics of PTHF–Mmt organophile nanocomposite. Arab. J. Chem..

[24] Belhouchat N., Zaghouaneboudiaf H., Viseras C. (2017). Removal of anionic and cationic dyes from aqueous solution with activated organo-bentonite/sodium alginate encapsulated beads. Appl. Clay Sci..

[25] Jin J., Tan Y., Liu R., Zheng J., Zhang J. (2019). Synergy Effect of Attapulgite, Rubber, and Diatomite on Organic Montmorillonite-Modified Asphalt. J. Mater. Civ. Eng..

[26] Solomon M.J., Almusallam A.S., Seefeldt K.F., Somwangthanaroj A., Varadan P. (2001). Rheology of polypropylene/clay hybrid materials. Macromolecules.

[27] Mcnally T., Murphy W.R., Lew C.Y., Turner R.J., Brennan G. (2003). Polyamide-12 layered silicate nanocomposites by melt blending. Polymer.

[28] Uno H., Tamura K., Yamada H., Umeyama K., Hatta T., Moriyoshi Y. (2009). Preparation and mechanical properties of exfoliated mica-polyamide 6 nanocomposites using sericite mica. Appl. Clay Sci..

[29] Liang Y., Ding H., Wang Y., Liang N., Wang G. (2013). Intercalation of cetyl trimethylammonium ion into sericite in the solvent of dimethyl sulfoxide. Appl. Clay Sci..

[30] Liang Y., Ding H., Sun S., Chen Y. (2017). Microstructural Modification and Characterization of Sericite. Materials.

[31] Gu C., Peng T., Sun H., Lv X., Luo L. (2012). Assembled Structure and Characterization of TiO_2_/Montmorillonite nanocomposites. J. Synth. Cryst..

